# Under the Mask: A Film on Tuberculosis at the Thai-Myanmar Border

**DOI:** 10.3389/fpubh.2022.795503

**Published:** 2022-04-21

**Authors:** Michele Vincenti Delmas, Mary Soan, Napat Khirikoekkong, Ko Ko Aung, Saw Ler Wah, Win Pa Pa Htun, Banyar Maung, Mavuto Mukaka, Phaik Yeong Cheah

**Affiliations:** ^1^Shoklo Malaria Research Unit, Faculty of Tropical Medicine, Mahidol University, Mae Sot, Thailand; ^2^Sermpanya (FilmAid) Foundation, Mae Sot, Thailand; ^3^Mahidol Oxford Tropical Medicine Research Unit (MORU), Faculty of Tropical Medicine, Mahidol University, Bangkok, Thailand; ^4^Nuffield Department of Clinical Medicine, Centre for Tropical Medicine and Global Health, University of Oxford, Oxford, United Kingdom; ^5^Nuffield Department of Population Health, The Ethox Centre, Oxford, United Kingdom

**Keywords:** Under the Mask, tuberculosis, community engagement, public engagement, film, Thai-Myanmar border, migrant health

## Abstract

In this paper, we describe the development of the film, “Under the Mask,” which follows the lives of three fictional characters who live on the Thai-Myanmar border as they journey from diagnosis of tuberculosis (TB) to completion of treatment. Under the Mask was filmed on location on the Thai-Myanmar border by local filmmakers and former refugee populations. Cast members were chosen from communities living along the border. This paper describes the script development process, filming, and screening in the community. We also report the findings from the pre- and post-screening questionnaires and post-film focus group discussions. A total of 77 screening events took place between March 2019 and March 2020 to 9,510 audience members in community venues such as village squares, temples and monasteries (*N* = 21), schools/migrant learning centers (*N* = 49), and clinics (*N* = 4). The pre-and post-screen questionnaires showed a significant gain in self-perceived TB knowledge on prevention, transmission, signs and symptoms, and related discrimination. Our findings from 18 post-screening focus group discussions conducted with 188 participants showed that there were improvements in knowledge and awareness of the disease and treatment, as well as in the awareness of stigma, and the burdens of tuberculosis on patients and their families.

## Introduction

The World Health Organization (WHO) declared that tuberculosis (TB) was a global emergency in 1993, making it the first infectious disease to be declared as such. In 2021, TB remains a major health problem (1), particularly in developing countries (2), and is one of the top 10 most lethal diseases worldwide. Over 10 million people contract TB annually, with a corresponding 1.3 million TB deaths, 45% of which occur in South-East Asia (1). In addition to the significant burden on the healthcare system, TB is often accompanied by severe economic and social consequences, a situation exarcebated by co-infection with HIV-AIDS and the increasing prevalence of multi-drug resistant TB (MDR-TB) (3, 4).

Myanmar and Thailand have a high burden of TB, including MDR-TB, and TB with HIV-AIDS co-infection. Indeed, both countries were among the 14 countries on the WHO's three high-burden country lists for TB, TB/HIV and MDR-TB for the period, 2016–2020 (1). MDR-TB, cross-border migration and border health issues are important barriers to ending TB in both countries.

TB in the population is worsened by specific population characteristics, such as poverty (5, 6), poor education (7), poor access to healthcare (8), and civil conflict (9). In addition, migrants are predisposed to contracting TB (10); their uncertain legal status often limits access to reliable health information and healthcare services, making them more at risk (11, 12). Many migrants also live and work in conditions that may contribute to the spread of MDR-TB due to many contacts, long journeys to and from work, and crowded living conditions (13, 14).

The Shoklo Malaria Research Unit's (SMRU) TB programme, currently funded by the Global Fund to Fight AIDS, TB and Malaria (previously funded by UKAid), provides free diagnosis and treatment services on the Thai-Myanmar border. SMRU is a field research site of the Bangkok-based Mahidol–Oxford Tropical Medicine Research Unit (MORU), which has its offices and laboratories in Mae Sot, Thailand, and clinics located on both sides of the border. SMRU has provided free humanitarian healthcare, including for mothers and children, and conducted health research since 1986.

The majority of the border population have low literacy and are “undocumented,” making them more vulnerable to poor health (15). Since the 1980s, political and militarized ethnic conflicts within Myanmar have forced hundreds of thousands of people, especially ethnic minorities, to take shelter, seek work opportunites and healthcare in Thailand, including in SMRU clinics. Thai nationals do not tend to access SMRU clinics as they can access government hospitals and have Universal Health Coverage.

The SMRU TB programme targets Karen and Myanmar migrants, and poor people from surrounding rural areas who face many barriers in accessing good quality healthcare and health information. Some patients come from as far as Yangon in Myanmar (14). In 2018 and 2019, SMRU screened 1,372 and 1,264 people for TB, of which 14.2% and 12.0% tested positive, respectively. On average, 80–90% of detected cases were enrolled for treatment under its TB programme, while others were referred for treatment with Myanmar health facilities.

SMRU has also developed a residential programme, “TB Village,” on each side of the border, where patients stay for the duration of their treatment (16). The TB Village can house ~160 patients at any given time, and is staffed by about 50 employees who are mainly Burmese and Karen, including five doctors. Rows of one-room dwellings accommodate patients, and accompanying family members are housed separately. Patients are provided shelter, medication, and food free-of-charge. In total, SMRU has ~300 in-patients and out-patients under their care at any given time.

Studies show that those with a low level of TB prevention and care knowledge have a greater chance of TB disease than those with a high level of TB prevention and care knowledge (17). Health education activities, pamphlets and other printed media have been used to promote TB awareness and health education, but in the context of the Thai-Myanmar border, these approaches have limitations. Low literacy, multiple ethnic languages, and hard-to-reach villagers means that innovative engagement approaches are needed to reach the target audience. In Southeast Asia, science-arts approaches such as folk songs (18), community drama (19–23), forum (24), and puppet theater (25), have been used for health education and to support health research. These blended science-arts events are typically science-themed events co-created by scientists and collaborating artists. Some benefits of this approach in relation to research in Southeast Asia have included facilitating understanding of disease (e.g., malaria), understanding of research, encouraging research participation and strengthening the ethics of research studies (19, 20, 23, 26, 27).

In response to the need to convey clear, accessible messages around TB and TB research to our target communities, the TB film project was created to supplement existing text-based messaging on TB and other efforts by local authorities and non-govermental organizations (NGOs). We set out to produce a full length, context-specific feature film designed to be brought to the community using “mobile cinema.” The aim of the project is to spread awareness of TB in the community SMRU serves, to encourage TB screening and early treatment, reduce stigma, and support treatment adherence for TB patients. The project was also an opportunity for our TB doctors and healthcare staff to better understand the experience and emotional journey of TB patients and their families to improve their own practice.

Using film to convey messages about TB is not new. Indeed, it is an approach that has been used since the early twentieth century, when a series of six, one-reel silent films were made by the National Association for the Study and Prevention of Tuberculosis (NASPT) in collaboration with the Edison Company: The Red Cross Seal (1910), The Awakening of John Bond (1911), Hope: A Red Cross Seal Story (1912), The Price of Human Lives (1913), The Temple of Moloch (1914), and The Lone Game (1915) (28). The visual narrative of these films made germ theory comprehensible, intelligible, and acceptable (28).

This paper describes the development of the Under the Mask film, the filming process, screening in the community, and the findings from the pre- and post-screening questionnaires and post-screening focus group discussions.

## Materials and Methods

### Project Team

In 2018, the SMRU TB and engagement teams commenced working with FilmAid Foundation (FAF), an NGO based in Mae Sot, on the TB film project, Under the Mask. Set up by FilmAid Asia (now operating under its Thai name, Sermpanya), FAF uses film and digital media to create accessible education and health information for migrant, refugee and other vulnerable populations. It also conducts media training courses to empower refugees to learn film-related skills to enable them to create films with stories, in their own voice and culture, which are then shared with their communities through interactive mobile cinema activities.

FAF was chosen to co-produce this film with SMRU/MORU due to their experience making impactful films using a participatory production approach, their experience in conducting mobile cinema events as well as their deep understanding of the Thai-Myanmar border context. In addition, FAF had collaborated with SMRU in 2016 to produce a short film to support a TB screening project in a refugee camp.

SMRU has a core engagement team with extensive experience in engaging with local communities on both sides of the border. The team have used multiple engagement approaches including consultation with community advisory boards (29–31), using science-art approaches (21), and participatory visual methods (15).

### Script and Narrative

The first step in the development of the script and narrative process saw the organization of a workshop with doctors and healthcare staff involved in the TB programme. The workshop was attended by 13 staff members who discussed the key messages, objectives, and target audience for this film project.

Real-life stories informed the content. To obtain these, the project team interviewed six TB patients undergoing treatment at SMRU TB clinics. In addition, the FAF team spent 6 months (February to August 2018) observing activities in the TB Villages. These observations, interviews with patients and healthcare staff, and patient testimonies informed the development of the film characters, emotions, narrative, and film script. The script was co-authored by FAF and the SMRU project team. The final story follows the lives of three fictional characters who live on the Thai-Myanmar border as they journey from diagnosis to completion of treatment.

The first character, U Tajar Min in his forties, has symptoms of TB but is reluctant to acknowledge it for fear of not being able to work. He uses various traditional medicines to try and cure himself before finally accepting that he must seek professional treatment. The second character, A Tun, in his 20s how, loses his job in a rubber plantation due to chronic back pain, and later discovers he has bone TB, a condition that few are aware of. The third character, Ma Zar Zar, in her 50s, is already having treatment in the TB Village, and faces serious issues of stigma from her family, including her own mother.

In the film, the doctors and healthcare staff explain TB, how to treat it, how to avoid reinfection, and what happens after treatment has been completed. For example, Dr. Banyar Maung, who plays himself, says “*you must make sure your room has fresh air and is kept very clean. You should eat regularly to keep your body strong and healthy,” “when a patient has completed their treatment successfully, we give them a certificate which confirms their sputum is clear and that they have tested negative for TB disease. Keep this certificate safely as it will reassure your family and community.”*

Verbal consent was obtained from all those involved in the workshops and interviews that informed the development of the film narrative, and all those that appeared in the film provided written consent.

### Film Crew, Cast Members, and Filming

The crew consisted of FAF-trained filmmakers from local communities and former refugee populations. Cast members were chosen from local SMRU staff (e.g., doctors, nurses, counselors) and the community living along the Thai-Myanmar border *via* auditions. There were 34 speaking characters and a total of 43 cast and background characters. These “actors,” including several who were illiterate, received TB information from healthcare staff, as well as acting coaching from FAF staff.

Scientific oversight was provided by TB doctors and researchers at SMRU, and engagement staff. Filming took place between September to December 2018 in the “TB Villages,” and in villages in the surrounding community.

### The Film

Initial rough cuts were reviewed by the project team. The near final versions were reviewed for scientific accuracy, context sensitivity and cultural appropriateness by SMRU staff.

The final product was a high quality, 75-min film in Burmese, with subtitles in English, Karen, and Thai. It was later dubbed in Karen. The film took its name, Under the Mask, from the fact that TB patients have to wear masks for the duration of their treatment, which can last for up to 20 months.

Approvals for the project, including filming, screening of the film, and evaluation were obtained from national, provincial and village level authorities on both sides of the border prior to the start of the project.

### Pre- and Post-screen Questionnaires and Statistical Analysis

All attendees at the film screening were given questionnaires in hard copy before the screening and again after the screening. They were asked to rank their knowledge on prevention, transmission, signs and symptoms of TB, and TB-related discrimination by choosing one of the following: “very little,” “quite little,” or “well enough,” before and after watching the film.

The comparison of self-knowledge was conducted using a chi-square test for trend. Statistical significance was declared at 5% significance level. The analysis was done using Stata 17 College Station, Texas 77845 USA.

### Post-screening Focus Group Discussions

After each film screening, participants were invited to join a post-screening focus group discussion for evaluation of the film and the film screening event, which was held either immediately after the film screening or the next day. Participants were not pre-selected for the focus groups based on a set of criteria. It was not possible to do so because we did not collect their demographic data. Rather, we had an open invitation to all who attended.

The focus group discussions were conducted in Burmese and Karen following a topic guide, by the TB team led by a trained TB counselor (KKA) experienced in facilitating focus group discussions. Interviews were transcribed and translated verbatim to English and manually coded. Coding was conducted using a combination of inductive and deductive approaches.

Verbal informed consent was obtained from all participants prior to focus group discussions. Written consent was not obtained because participants, who were primarily undocumented migrants, could be put at risk by existence of a paper record (15). For this reason too, attendees of events were told that we would not collect any personal information (e.g., name, age, gender, location, occupation).

## Results

### Film Screening

The film premiered in the SMRU TB clinic in Koko village in Myanmar, on World TB Day on 22 March 2019 to ~300 audience members. Subsequently, a series of community screening or mobile cinema events were organized by the SMRU and FAF teams, in collaboration with village and community leaders, and school principals. Screening events involved transporting equipment from FAF and SMRU offices to the screening venue. The mobile cinema team consisted of technical persons, SMRU TB team and staff from FAF.

A total of 77 community screening events took place between March 2019 and March 2020 to 9,510 audience members in community venues such as village squares, temples and monasteries (*N* = 21), schools/migrant learning centers (*N* = 49) and clinics (*N* = 4).

Each screening was followed by an hour-long health discussion with the SMRU TB team. These health discussions focused on TB whereas the focus group discussions described above were for evaluation of the film and film screening event. The health discussion included topics such as where to get screened for TB, how to recognize symptoms, and how to prevent getting TB. For evening film showings, these discussions were conducted the following day. Audience members, who sometimes included recovered TB patients, took the opportunity to ask questions, share stories and experiences.

Film screenings took place in the evenings after villagers have returned from work, and school screenings took place during school hours. Participation in these events was free. Snacks and beverages were provided.

In addition to screening events in the community, the film was shown at various events and conferences around the world, targeting TB and health stakeholders such as journalists, community representatives, public health researchers, research funders, governmental and NGO partners (e.g., International Organization for Migration, Thai Border Consortium). These additional screenings reached 747 audience members. Some of these events included a post-show Q&A with the director and project team members. See Table 1 for details. Many more people have watched the film on YouTube, where it is freely accessible (https://youtu.be/kxKHFxcFeJ8).

**Table 1:** Details of Under the Mask screenings to TB stakeholders.

**Date**	**Venue; occasion**	**Type of audience**	**Number (approx.)**
24 March 2019	Shwe Ko Ko township, Myawaddy, Myanmar; worldwide premier	Local authorities, villagers	300
3–4 April 2019	Kickstart Art Summer School, Mae Sot, Thailand; in conjunction with another TB engagement project, Imaging In and Expressing Out. Children attending the Kickstart art summer school produced piece of drawing reflecting their feelings and impressions about the impact of TB on patients after watching the movie.	Children (12 to 18 years old)	30
6 May 2019	Ko Ko TB clinic, Ko Ko, Myanmar	TB patients, staff in TB clinic	Staff: 17 Patient and caregivers: 70 (number of patient and care givers are an estimation, please note that some patients also have their family living at the clinic).
14 June 2019	Child Development Center (CDC), Mae Sot, Thailand	Student High school and post-high school students Aged 16 to 25 (some audiences are other visitors in the area)	78
17 June 2019	Bangkok Screening Room, Bangkok, Thailand; Bangkok premier followed Q&A with director and producers	Media personnel, funders, NGOs such as The Border Consortium (TBC), Raksthai Foundation, and International Rescue Committee (IRC)	40
19 August 2019	Vientiane, Laos; Global Health Bioethics Network (GHBN) annual conference	Bioethicists, biomedical researchers	50
2 September 2019	The Foreign Correspondents' Club of Thailand, Bangkok, Thailand; followed Q&A with director and producers	Foreign correspondents in Thailand	10
3 September 2019	MORU office, Bangkok, Thailand	TB stakeholders working Thailand e.g., IOM, World Vision, and Raksthai Foundation	22
23 November 2019	National Harbor, USA; American Society of Tropical Medicine & Hygiene Annual Meeting	Tropical medicine researchers, attendees at ASTMH	88
28 November 2019	Wellcome Trust, London, UK	Wellcome Trust employees	12
4 February 2020	Shoklo Malaria Research Unit, Mae Sot, Thailand	Senior University of Oxford and Wellcome Trust staff and international experts on tropical medicine; as part of the quinquennial review of MORU	30
Total			747 (estimation)

For screenings in the community, sessions were evaluated quantitatively using pre- and post-test questionnaires, followed by focus group discussions after the screening. The questionnaires evaluated audience's self-perceived knowledge before and after the screening on prevention, transmission, symptoms, treatment, stigma and discrimination.

### Pre- and Post-screen Evaluation

A total of 5,761 and 5,803 people completed the questionnaire before and after watching the film. These figures represent 60.1 and 61.0% of attendees of the film screening. The results showed a significant gain in self-perceived knowledge of each of the categories: TB knowledge on prevention, transmission, signs and symptoms of TB, and TB-related discrimination. There was a statistically significant association between: knowledge about prevention or knowledge about transmission or knowledge about signs and symptoms for TB or knowledge about discrimination and test period, whether pre-test or post-test, *p* < 0.0001 for all tests. The proportions having well enough knowledge in the post-test period were very high relative to those observed in the pre-test period for all the knowledge parameters of interest (Table 2). The prevalence of “well enough” knowledge increased from 26.3 to 52.6 %; 11.9 to 41.6%; 10.1 to 35.1%; and 6.4 to 14.3% between pre-test and post-test, respectively for knowledge about prevention, knowledge about transmission, knowledge about signs and symptoms for TB or knowledge about discrimination. Table 2 shows the self-perception of knowledge of TB pre- and post-film screening.

**Table 2:** Self-perceived knowledge of TB by pre- and post-film screening.

**Variables**	**Total** ***N*** **= Respondent who answers** **the questions**		
	**Pre-test** ***n/N* (%)**	**Post-test** ***n/N* (%)**	***P*-Value** **(trend test)**
Knowledge about prevention	5,754/5,754 (100)	5,772/5,772 (100)	<0.0001
Very little	2,226/5,754 (38.7)	698/5,772 (12.1)	
Quite little	2,014/5,754 (35.0)	2,038/5,772 (35.3)	
Well enough	1,514/5,754 (26.3)	3,036/5,772 (52.6)	
Knowledge about transmission	5,738/5,738 (100)	5,773/5,773 (100)	<0.0001
Very little	2,101/5,738 (36.6)	595/5,773 (10.3)	
Quite little	2,952/5,738 (51.4)	2,778/5,773 (48.1)	
Well enough	685/5,738 (11.9)	2,400/5,773 (41.6)	
Knowledge about signs and symptoms for TB	5,761/5,761 (100)	5,803/5,803 (100)	<0.0001
Very little	3,011/5,761 (52.3)	1,009/5,803 (17.4)	
Quite little	2,167/5,761 (37.6)	2,757/5,803 (47.5)	
Well enough	583/5,761 (10.1)	2,037/5,803 (35.1)	
Knowledge about discrimination	5,751/5,751 (100)	5,762/5,762 (100)	<0.0001
Very little	3,035/5,751 (52.8)	1,871/5,762 (32.5)	
Quite little	2,349/5,751 (40.8)	3,066/5,762 (53.2)	
Well enough	367/5,751 (6.4)	825/5,762 (14.3)	

### Findings From Focus Group Discussions

The following section describes the findings from the focus group discussions conducted with audience members after the film screening. A total of 18 focus group discussions were conducted with 188 adult participants between 23 May 2019 and 3 February 2020.

Our findings demonstrated improvements in the knowledge and awareness of TB disease and treatment, as well as in the awareness of stigma, and the burdens of TB on patients and their families. Audience members endorsed the film as a favorable engagement approach.

Each theme is discussed in turn below.

#### Improving Knowledge and Awareness of TB Disease and Treatment

By watching this film, many villagers expressed that they have learned a lot about TB, “*we did not know there are different kinds of TB until we watched this movie*.” In the film, two characters had pulmonary TB and one had bone TB. They also said that that they now know what they should do and where to go if they think they have contracted TB.

Village chiefs who helped facilitate the community events were key to refer villagers, many of whom had questions following the events, to the appropriate place to get TB information and diagnosis. This was especially important to those living on the Myanmar side of the border, as they did not know where to seek help for TB. Villagers said they learned what facilities are available to them, including the SMRU TB clinic, which they thought only treated malaria.

Villagers gained knowledge they did not know before, such as the symptoms of TB, where to get tested, how it is transmitted and how to support TB patients. Some of the symptoms, such as coughing up blood, was not known prior to watching the film. By watching this film, some villagers learnt that TB is one of the most fearful diseases, but can be cured by taking drugs until treatment is completed. According to one villager, “*after watching this movie, there are two things coming in my mind. One thing is I need to be aware more about TB disease before I get TB disease. Second thing is, if I have TB signs and symptoms, I need to go to the TB clinic as fast as I can.”* This is an important message because many villagers prefer to get treated by traditional healers rather than going to a modern clinic or hospital as illustrated by one of the characters, U Tajar Min.

Villagers also expressed that they were not aware that once a patient has completed their TB treatment, they are given a certificate or equivalent documentation to certify their treatment completion. Such documents were thought to be useful to show to neighbors and employers.

#### Improving Awareness of Stigma, and the Burdens of TB on Patients and Their Families

Film screenings had a positive emotional impact on patients and the community, as the issues and burdens, including stigma, that they faced were acknowledged and discussed. Through the film, the voices of TB patients, their families and health care staff were heard.

Audience members confirmed that the stigma and discrimination against TB patients and their families still exists in the community, for example, friends and relatives will avoid talking to TB patients. Some said they sympathized with the character, U Tajar Min, because he and his family are discriminated against by the people around them. They said the scenes in the film reflect the reality*: “The scene where a TB patient was not allowed to drink water from a communal water jar made me feel sorry for her, and I now understand the patient's feeling.”*

Some villagers expressed fear of getting tested for TB or of losing their job, like one character in the film. Most of them said they feel nervous to get tested*:“A lot of people can relate to one character, a patient's husband. He was afraid to test for TB, for he feared that he would have to undergo 6 months TB treatment, which is a long time.”*

A villager said that if they have to feed TB patient, they will cover their mouth with a mask and explain to the patient that it is not a discrimination, but rather a prevention measure to avoid getting TB from them.

#### Film as an Engagement Approach

The film was described as entertaining, and made learning and understanding of TB more interesting compared to conventional health education methods. Villagers especially enjoyed watching their friends and family, and healthcare staff as “actors” in the film.

“*Health education with the movie is more effective than verbal sessions, because we can memorize a lot and share what's in the movie…pamphlets are not very effective, as most villagers can't read or write.”*

“*Verbal health campaign is boring but watching movie like this is more interesting, I can still remember some of them after watching.”*

They also enjoyed the humor in the film, and the affection that develops that bonds all the characters together, for example, when A Tun gets a crush on one of the healthcare workers. The scenes related to this were crafted delicately due to the issue of staff-patient relationships, but it was thought necessary to inject humor into a film about a serious subject. These scenes were really about demonstrating the care of healthcare staff toward their patients, which contributes to TB patients' compliance to treatment and recovery.

## Discussion

### Engagement Using Film

The method we took to develop the film has been described as “participatory visual methods (PVM)” approach. The term PVM describes an range of facilitated processes that support participants to produce or co-produce with others their own images or visuals such as film, photos, drawings and paintings (32). PVM has been shown to encourage patients and research participants to express themselves in ways that are not made possible by traditional qualitative methods such as formal interviews or focus group discussions (33). PVM can offer participants visual ways of articulating honest information that may be challenging to communicate because of language barriers, topic sensitivity or feelings of *kreng-jai* (Thai) or *arr-nar* (Karen/Burmese) which is a familiar cultural tendency in this part of the world. *Kreng-jai*/*arr-nar* is understood as “the desire to be self-effacing, respectful, humble, and extremely considerate, as well as the wish to avoid embarrassing others or intruding or imposing on them” (34). That means that sometimes, patients and study participants are reluctant to tell doctors and researchers how they really feel because they are embarrassed or do not want to inconvenience them.

In our project, TB patients and carers told us their stories so that they can be told *via* fictional characters in the film. We learnt things that we had not previously appreciated especially the extent of the stigma they faced from family and community members.

The project engaged with TB stakeholders at different levels. In the initial development process of the film and narrative, in-depth interactions with TB patients and healthcare staff enabled the project team, cast and crew to gain a deep understanding of their experiences such as the challenges of living with TB both as a patient and as a family member or carer of a TB patient. These local stories and testimonies were an opportunity for researchers and doctors to listen and learn, and helped embed TB patients and healthcare staff voices in the film. This learning was further enhanced during script development, and later, filming. Through the roles they played in the film, members of the cast more closely understood the experience of being a TB patient or carer.

On location filming and involvement of a large number of cast and crew members also raised awareness of TB in the local area. The non-professional cast and crew members learnt new skills. The co-production of the film by SMRU staff, patients, clinic staff and FAF strengthened relationships between the SMRU team and healthcare stakeholders in the region.

From the pre- and post-screening questionnaires and focus group discussions with audience members, we found that the film and the accompanying post-film health discussion improved knowledge and awareness of TB, as well as awareness of stigma and the burdens on TB patients and their families. We are hopeful that this awareness leads to behavior change around stigma and discrimination, as well as encouraging those who are at risk to seek treatment early.

At the time of writing, nearly 10,000 villagers along the Thai-Myanmar border had watched the film at mobile screening events, with many more having watched *via* other channels. The screenings reached illiterate audiences that traditional modes of communications do not usually reach. The film, now freely available, has the potential to reach a wide range of international audiences, with or without any facilitation by a TB expert.

The film has become popular among villagers living along the Thai-Myanmar border. The original version is in Burmese and dubbed in Karen, the language spoken in the border area. Villagers, village leaders, and healthcare workers have endorsed using the film as an engagement approach, particularly due to its entertainment value above and beyond the educational value. TB stakeholders intend to use the film when teaching medical students and healthcare workers about TB and we have shared the film with colleagues conducting TB research and teaching TB to medical students. Science-arts collaborations have been popular in engagement around TB. For example, in South Africa, the film, The Lucky Specials, explores issues of drug adherence and the risks of MDR TB while the Eh!woza (Hey! Come with us) project saw scientists engage with young people to produce short films about experiences of TB within their communities (35).

### Lessons Learnt

Hearing patient narratives and participating in the discussion sessions moderated by the TB team after the film screenings has been an effective way for researchers and healthcare workers to listen and learn from the community. Clinic staff who played characters in the film experienced the lives of a TB patient or a carer at a much deeper level. The questions and comments from the screenings in communities helped the team to understand that TB is still very unknown and stigma is still very prevalent. We also confirmed with our previous findings in a qualitative study that migrants experience particular barriers to seeking diagnosis and treatment due to their legal status, transportation challenges, and lack of finances (12). To come to the clinic for testing, daily wage migrants lose the day's income (12, 15). Undocumented migrants fear being stopped by the police, an incident that may see them face deportation (14, 15, 30).

From this learning, we have increased efforts to encourage villagers to come for TB testing at our clinics. Because some migrants have difficulty reaching our clinics due to transport and financial constraints, we have also set up mobile TB screening initiatives to bring care to them.

The SMRU TB counseling team has been using some of the film's scenes in the counseling sessions, such as a scene revolving around a TB patient losing her rented accommodation because her landlord did not want the her to return to the accommodation, even after her recovery. This has been particularly useful for facilitating discussions on learning how to cope with stigma around TB. Stigma around TB is prevalent (14, 36), and coping with stigma is important because reducing stigma at the community-level is challenging and takes more time.

### Strengths and Limitations

In terms of strengths, the film project was the first of its kind for the Thai-Myanmar border population, and has provided engagement practitioners here with much food for thought for future engagement work such as finding alternative ways of engaging with the community taking into account their low literacy and multiple languages used, and limitations in travel. We found that the “mobile cinema” approach was an effective way to engage audiences. In our 77 mobile cinema events, only one person left before the end of the film because he had to guard his crops from wild elephants and other animals.

The film was viewed by audiences as entertainment rather than as an educational film, therefore it has the potential to reach wider audiences. We intentionally limited the purely informational part of the film, but the post-film discussion reinforced the TB messages we wanted to convey.

In addition to spreading knowledge and awareness about TB, the film had other positive impacts on those involved and in the surrounding communities, providing jobs and learning opportunities for the villagers.

The project has already led to more arts-science initiatives by the SMRU engagement team—in TB, as well as other diseases, such as malaria and COVID-19. We have made shorter films for use in other settings, i.e., where mobile cinema events are not possible.

One limitation is that the film was not co-created with patients, unlike TB participatory educational films, such as those evaluated recently in the United States (37). Instead, it was informed by patient testimonies and experiences of TB doctors, and the script was co-developed by TB researchers and local filmmakers, who had a deep understanding of TB and the Thai-Myanmar border context. The director, a refugee from Myanmar, and his team, along with the local cast, made the film authentic; no professional actors were involved. The film was made in Burmese and later dubbed in Karen, but some people spoke other Karen dialects.

Additionally, while the film covered many aspects of TB, it did not discuss MDR-TB or TB/HIV, both of which are becoming increasingly important (1). We will address these in future projects.

Lastly, approximately only 60% of the audience completed the pre- and post-film questionnaires as many were illiterate. This may have caused the results of the questionnaires to be less reflective of the reality. As for the focus group discussions, only people who could spare the time attended the sessions. But we had a larger number than expected (118 participated).

### Ethical and Practical Challenges

Under the Mask told the stories of three fictional characters living on the Thai-Myanmar border. The actors were not TB patients but recruited from the community. For example, the “actor” playing one of the TB patients worked as construction worker on a large building development near the TB Village. The writers made every effort to ensure that the characters in the film did not resemble real-life patients.

We obtained verbal but not written consent from participants of the focus group discussions because many were undocumented migrants, and a handful were recovered TB patients. Undocumented migrants are not allowed to travel freely especially outside “safe hours” and “safe zones” (30). The existence of a paper record could put them at risk of being fined, arrested or deported to their home country (15). For this reason too, no attendee details were obtained during the film screening or focus group discussions. We therefore do not have the demographic details (e.g., age, gender, occupation) of who attended the screening events or focus group discussions.

The challenges of making a film of this nature without a professional cast should not be underestimated. Training community actors, some of which were illiterate, while rewarding took a lot of time. Another challenge was filming on location at the TB clinics and surrounding villages. There was a lot of background noise as there was a large logging entity with saws running throughout the day. In addition, filming on bamboo floors, which are typical of the houses in these villages, was difficult as just one footstep could move the camera. The community screening events was labor intensive and had to be conducted in the evenings which meant staff had to work extra hours.

## Conclusions

Under the Mask was the first of its kind for the Thai-Myanmar border population, and has provided engagement practitioners with much food for thought for future engagement work. There is a need to find innovative ways to spread awareness of TB, to encourage TB screening and early diagnosis and treatment, to reduce stigma, to encourage positive health seeking behavior and support treatment adherence for TB patients. There is also a need for TB doctors and researchers to embed voices of TB patients and communities affected by TB in the management of TB patients and future conduct of TB research.

We found that the “mobile cinema” approach, which brought the film to rural communities, followed by discussion about the film and TB, was an effective way to engage audiences in rural communities on the Thai-Myanmar border. The “mobile cinema” approach brought the film and associated health discussions to the community such villages or schools, rather than asking the community to go to another venue they may not be familiar with. The latter approach to public engagement has been criticized because it tends to miss reaching to some subsets of the community and for “preaching to the converted,” whereby attendees are those already engaged within the scientific field (38).

The pre-and post-screen questionnaires and focus group discussions showed that there were self-reported improvements in knowledge and awareness of the disease and treatment, as well as in the awareness of stigma, and the burdens of tuberculosis on patients and their families. The project was also an opportunity for our TB doctors and healthcare staff to listen to TB patients and their families, so that they can improve their own practice.

## Data Availability Statement

The raw data supporting the conclusions of this article will be made available by the authors, without undue reservation.

## Ethics Statement

Ethical review and approval was not required for the study on human participants in accordance with the local legislation and institutional requirements. Written informed consent for participation was not required for this study in accordance with the national legislation and the institutional requirements.

## Author Contributions

MD and MS conceived and oversaw the project. MD, MS, and PC were producers of UTM and SL was the director. MD designed the evaluation forms. KA and MD conducted the surveys and focus group discussions. MM, KA, and NK conducted the analyses of the pre- and post-screen questionnaires and data from the focus group discussions. WH and BM provided oversight of the project in the TB clinics. PC raised the funding and wrote the first draft of the paper. All authors reviewed the manuscript and approved the final version.

## Funding

This work was supported by Wellcome Trust awards (106698 and 220211/A/20/A, and 096527).

## Conflict of Interest

The authors declare that the research was conducted in the absence of any commercial or financial relationships that could be construed as a potential conflict of interest. The handling editor is currently organizing a Research Topic with one of the authors PC.

## Publisher's Note

All claims expressed in this article are solely those of the authors and do not necessarily represent those of their affiliated organizations, or those of the publisher, the editors and the reviewers. Any product that may be evaluated in this article, or claim that may be made by its manufacturer, is not guaranteed or endorsed by the publisher.
